# Clinical Effect of Bacterial Suspension in Patients with Moderate-to-Severe Allergic Conjunctivitis

**DOI:** 10.3390/jcm15155962

**Published:** 2026-07-30

**Authors:** Israel Casanova-Méndez, Guillermo A. Quintana-Mexiac, José L. Alcalá-Gallegos, Henry Velazquez-Soto, Lorenzo Islas-Vázquez, Michelle Pacheco-Quito, Concepción Santacruz-Valdés, María C. Jiménez-Martínez

**Affiliations:** 1 Department of Non-Ophthalmological Emergencies, Instituto de Oftalmología “Conde de Valenciana”, Ciudad de Mexico 06800, Mexico; israel.casanova@condecentro.org (I.C.-M.);; 2 Department of Immunology and Research Unit, Instituto de Oftalmología “Conde de Valenciana”, Ciudad de Mexico 06800, Mexico; 3 Pharmacovigilance Unit, Instituto de Oftalmología “Conde de Valenciana”, Ciudad de Mexico 06800, Mexico; 4 Department of Cornea and Refractive Surgery, Instituto de Oftalmología “Conde de Valenciana”, Ciudad de Mexico 06800, Mexico; 5 Department of Biochemistry, Faculty of Medicine, National Autonomous University of Mexico, Ciudad de Mexico 04510, Mexico

**Keywords:** allergic conjunctivitis, bacterial suspension, immunomodulation, IL-10, regulatory B cells, tear film, pediatric allergy, ocular surface inflammation

## Abstract

**Background/Objectives**: Allergic conjunctivitis (AC) is a frequent inflammatory ocular surface disease that significantly affects quality of life, particularly in children. Current treatments mainly provide temporary symptom relief and often require prolonged use. Bacterial suspensions have emerged as potential immunomodulatory treatments for other allergies but have yet to be completely explored for ocular allergies. Our study’s objective was to describe the clinical, ophthalmological, and quality-of-life changes in patients with AC treated with a bacterial suspension (BS) as complementary therapy. **Methods**: A before-and-after open, non-controlled clinical study was conducted in five children aged 7 to 9 years with a diagnosis of moderate-to-severe persistent allergic conjunctivitis and negative skin prick test results. Clinical ocular signs and symptoms, quality of life, and changes in CD19^+^IL-10^+^ cells were assessed. **Results**: After 90 days of BS treatment, reductions in allergic symptoms, including itching, light sensitivity, and burning, were observed, along with a marked reduction in ocular inflammation. An evaluation of quality-of-life revealed improvements across all domains and an increase in CD19^+^IL-10^+^ cells. **Conclusions**: BS therapy demonstrated favorable clinical and immune-modulatory effects in children with AC, supporting its potential as a promising complementary therapeutic option.

## 1. Introduction

Allergic conjunctivitis (AC) is an inflammatory disease of the ocular surface that develops in previously sensitized individuals following exposure to one or more allergens [1]. Epidemiological studies have reported that ocular allergy symptom prevalence ranges from 6% to 30% of the population, representing a considerable public health burden. Furthermore, in recent decades, this condition has been shown to cause ophthalmological impairment and to deteriorate patients’ quality of life by interfering with school, work, and social activities [2,3,4].

To improve clinical management, various research groups have characterized acute AC and developed international treatment reports and guidelines that recognize the importance of diagnosis based on clinical signs and symptoms [5,6]. Although symptomatic therapy is well established, a key limitation has been identified: antihistamines, mast cell stabilizers, and nonsteroidal anti-inflammatory drugs provide only transient relief, with symptoms recurring once treatment is discontinued [4,5,6]. This aspect of symptomatic treatment often leads to prolonged and chronic medication use, increasing the risk of adverse effects, decreasing therapeutic adherence, and negatively impacting patients’ quality of life. Several studies have documented how this situation can cause anxiety, stress, and significant emotional impairment [3,7,8]. Given these limitations, there has been growing interest in immunomodulatory therapies, particularly allergen-specific immunotherapy (AIT); these approaches require prior identification of the clinically relevant allergen through in vivo or in vitro detection of specific IgE [9,10]. In this context, several studies have shown that AIT reduces symptoms, medication requirements, and combined symptom–medication scores during treatment, with some evidence suggesting that symptom-related benefits may be sustained after treatment cessation [11]. However, in patients with ocular allergy, it is well known that the triggering allergen cannot always be identified through conventional skin testing [12]; in selected cases, conjunctival challenge testing may therefore be helpful in identifying the clinically relevant allergenic source [13]. Even after identifying the allergen at the ocular surface, evidence regarding AIT use guided by allergen selection through conjunctival challenge testing nevertheless remains lacking.

Further research on immunomodulatory strategies in patients with ocular allergy is therefore needed to expand therapeutic options, especially in difficult-to-control cases. In this context, several clinical trials have demonstrated that bacterial therapies may reduce symptoms and recurrence rates in allergic diseases such as asthma, allergic rhinitis, and dermatitis [14,15,16]; however, these therapies have not been fully explored in the context of ocular allergy.

In line with this evidence, our research group has previously demonstrated that a commercially available bacterial suspension can modulate the immune response by promoting regulatory B cell (Breg) activation, supporting its potential role as an immunomodulatory approach in allergic diseases [17]. The aim of this study was thus to evaluate a potential therapeutic strategy for patients with moderate-to-severe ocular allergy who had negative skin prick test results for identifiable triggering allergens and showed insufficient clinical response to standard ophthalmic treatment.

## 2. Materials and Methods

### 2.1. Patients

**Figure 1:**
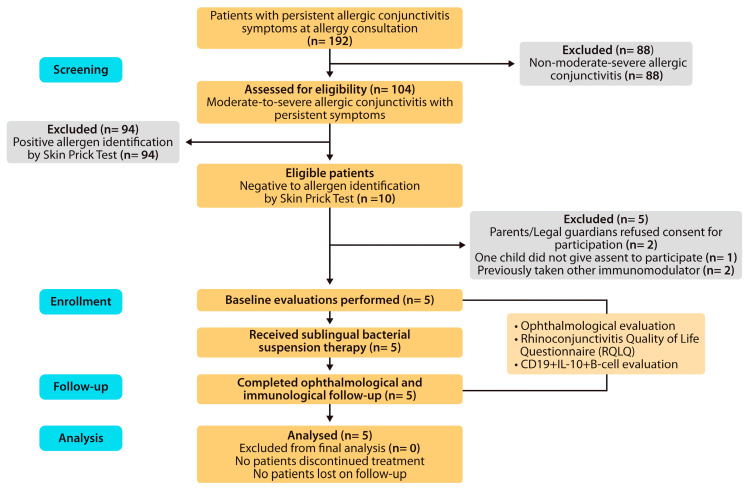
Study flow diagram.

The patients included in this study were five children (one girl and four boys) with moderate-to-severe AC, defined as ocular symptoms present more than 4 days per week for at least 4 consecutive weeks, according to the Consensus Document on Allergic Conjunctivitis (DECA) [18] (Figure 1). Clinical severity was evaluated by both an ophthalmologist (cornea specialist) and an allergist to ensure consistent diagnostic criteria. Patients with mild allergic conjunctivitis, those with allergens identified by skin prick testing, and those who had received previous treatments capable of modifying the immune response were excluded. Only patients with moderate-to-severe AC who had received more than three to six months of standardized ophthalmological treatment with poor clinical improvement were invited to participate.

Since the study population consisted exclusively of pediatric patients, which are considered a vulnerable population, written informed consent was first obtained from their parents or legal guardians, followed by assent from the children. The study was conducted in accordance with the Declaration of Helsinki and approved by the Institutional Review Board, including the Biosafety, Scientific, and Ethics Committees of the Institute of Ophthalmology “Conde de Valenciana” Foundation, in accordance with current Mexican regulations. The study was registered under the following approval numbers: CI-046-2016, CB-046-2016, and CEI-2016/10/03.

Once consent and assent were obtained, all patients underwent ophthalmological and immunological evaluations, and participants with parents or legal guardians completed a standardized quality-of-life questionnaire (RQLQ) to assess the subjective impact of treatment (in a Mexican Spanish-validated translation) [19,20] before and after three months of BS treatment.

### 2.2. Study Design

Our study was a quasi-experimental, open-label, before-and-after design.

### 2.3. Clinical Assessment Tools

#### 2.3.1. Symptom Score

A clinical evaluation of the symptoms included itching, tearing, photophobia, foreign body sensation, and burning. A numerical value was assigned to assess occurrence frequency: 0—no symptoms; 1—symptoms present once a week; 2—symptoms present 1 or 2 days a week; 3—symptoms present 3 or 4 days a week; 4—symptoms present 5 or 6 days a week; and 5—symptoms present every day of the week.

#### 2.3.2. Ophthalmological Score

The primary outcome was ophthalmological improvement. Clinical signs were evaluated by a corneal specialist, who used a 0–4 Likert scale to quantify the severity of each finding. The variables analyzed included conjunctival hyperemia and swelling, as well as tarsal conjunctiva inflammatory response.

Conjunctival hyperemia and swelling were graded according to the intensity of redness, edema, and the presence or formation of folds or plica in the conjunctival sac fundus. A score of 0 was assigned when no hyperemia or edema was present. A score of 1 corresponded to mild hyperemia (1+ to 2+) with approximately one-third conjunctival edema of a pink appearance, without plica formation in the conjunctival sac fundus. A score of 2 was assigned for moderate hyperemia (2+ to 3+) with approximately two-thirds conjunctival redness and edema and/or slight plica formation in the fornix. A score of 3 corresponded to moderately severe hyperemia (>3+) with more than two-thirds conjunctival edema and localized engorgement of the ciliary vessels, accompanied by moderate plica formation in the sac fundus. Finally, a score of 4 was assigned for severe hyperemia (>3+) with generalized engorgement of the ciliary vessels and severe plica or conjunctival folding in the sac fundus.

In the tarsal conjunctiva, the inflammatory response and presence and extent of papillae were evaluated, ranging from minimal papillary hyperplasia to macropapillae with fibrosis or symblepharon formation. A score of 0 was assigned when no papillary hyperplasia or follicles were observed. A score of 1 corresponded to papillae involving less than one-third of the tarsal conjunctiva, with a size of approximately 0.3 mm and visible, uniformly distributed tarsal vessels. A score of 2 was assigned for moderate papillary involvement (one- to two-thirds of the tarsal conjunctiva), with papillae measuring 0.3–0.5 mm and thin but visible tarsal vessels. A score of 3 corresponded to the presence of cobblestone papillae, involving more than two-thirds of the tarsal conjunctiva, with papillae approximately 0.75 mm in size, with or without fibrosis, and irregular tarsal vasculature. Finally, a score of 4 was assigned for large papillae (>0.75 mm) with fibrosis or macropapillae, extrusion, and possible fornix foreshortening (symblepharon), or a generalized pale appearance of the tarsal conjunctiva with absence of normally visible tarsal vessels.

#### 2.3.3. Schirmer Test

A strip of millimeter-scale paper (AMCON Tear Flo Test Strips Nomax, Inc., St. Louis, MO, USA) was placed on the lower eyelid of each eye. Both strips were applied simultaneously and removed 5 min after application. The paper’s moisture content was then measured, and the result reported in millimeters.

#### 2.3.4. Tear Break-up Time (TBUT)

The procedure was performed using fluorescein strips and by applying a drop of tetracaine (Bio Glo Fluorescein Sodium Ophthalmic Strips, HUB Pharmaceuticals, Cucamonga, CA, USA) to the lower conjunctival sac. The patient was asked to blink several times to distribute the dye throughout the tear film. The time elapsed from the last blink until the appearance of the first dry spot was then observed under a slit lamp with a cobalt blue light filter.

#### 2.3.5. Quality-of-Life Questionnaire on Rhinoconjunctivitis (RQLQ)

Patients with parents or legal guardians responded to each of the 28 items on the 7-domain questionnaire on a scale from 0 (Not at all bothersome) to 6 (Very bothersome). The total score was calculated as the mean of all item scores. Scores for each of the 7 domains (activity limitation, sleep problems, nasal symptoms, eye symptoms, non-nose/eye symptoms, practical problems, and emotional functioning) were obtained from the mean score of the items within each domain. Scores for individual items were also obtained. The primary outcome measure was a change from baseline in the overall, domain, and individual item scores of the RQLQ of at least 0.5 (minimum important difference [MID]). The degree of quality-of-life impairment was defined as follows: less than −0.5 was considered unfavorable, −0.5 to 0.5 was considered no effect, and greater than 0.5 was considered favorable [19,20].

### 2.4. Treatment

#### 2.4.1. Conventional Standardized Treatment

All included subjects had shown an inadequate clinical response despite receiving conventional ophthalmologic treatment for at least three to six months preceding study enrollment. Throughout the follow-up period, all patients continued the same conventional ophthalmologic treatment, consisting of olopatadine 0.2% ophthalmic solution (Patanol), one drop in each eye once daily, and lubricant eye drops (Systane), one drop in each eye every six hours, for three months (Alcon Laboratories, Inc., Fort Worth, TX, USA).

#### 2.4.2. Immunomodulator Treatment

All patients received conventional treatment plus BS administered as 2 sublingual drops daily (20 × 10^6^ killed whole bacteria, Polivacc, IPI ASAC, Ciudad de Mexico, Mexico) for 90 consecutive days, according to the manufacturer’s recommended dosing regimen. Treatment adherence was monitored through a daily log in which each patient recorded the administered dose and storage conditions throughout the study period. Baseline evaluations were performed before the addition of bacterial suspension therapy.

### 2.5. Adverse Event Evaluation

Although BS is considered a safe immunomodulator that has mainly been used for infectious diseases or respiratory allergies in children, in the present study, its use focused on evaluating potential clinical changes associated with ocular allergy. Thus, at each follow-up visit, directed questioning was conducted to identify known adverse events (AEs). Causality was assessed using Naranjo’s algorithm [21], and AEs were reported in accordance with Mexican regulatory requirements [22]. Codification was performed using the Medical Dictionary for Regulatory Activities (MedDRA) [23] (Spanish version 29.0).

### 2.6. CD19+ IL-10+ Cell Evaluation

To evaluate changes in the percentage of CD19^+^ IL-10^+^ cells before and after treatment, peripheral blood samples were labeled with mouse monoclonal antibodies conjugated to allophycocyanin (APC) anti-human CD19 and phycoerythrin (PE) anti-human IL-10 (BD Biosciences, San Jose, CA, USA). Samples were analyzed on a BD FACSVerse™ flow cytometer using FACSuite software version 1.0.5.3841, acquiring 10,000 events per sample. The identification strategy included single-cell selection using FSC-A vs. FSC-H, delineation of the lymphocyte population by size, identification of CD19^+^ cells, and finally, evaluation of intracellular IL-10 expression within this population.

### 2.7. Statistical Analysis

The results described in this work use median and interquartile rank (IQR), according to the data distribution. Comparisons were performed using a Wilcoxon signed-rank test for paired samples, with a *p*-value < 0.05 considered statistically significant. The PRISMA 10.6.1 GraphPad software was used.

## 3. Results

### 3.1. Participants

The study population consisted of five patients, whose demographic characteristics are presented as medians and interquartile ranges (IQRs) in Table 1 and Table S1.

### 3.2. Ophthalmological Evaluation

Ocular pruritus and a gritty sensation were present in all cases before the intervention, occurring 3 to 4 days per week, and completely resolved after treatment. Photophobia and a burning sensation were present in four patients before treatment; the first occurred 1 to 6 days per week and resolved in four of five cases at the end of BS treatment; the second occurred 1 to 4 days per week and completely resolved in all cases. Tearing was present in three patients and occurred 3 to 6 days per week; only one patient reported tearing at the end of BS treatment.

According to the DECA, all patients had persistent AC; one patient was classified as having moderate AC, while four patients presented with severe AC at baseline. After BS treatment, four patients were classified as having mild AC, and all participants were classified as having intermittent AC. All five patients presented with mild-to-moderate signs of ocular inflammation at baseline, and after BS treatment, hyperemia, conjunctival swelling, and inflammatory response of the tarsal conjunctiva diminished significantly (*p* = 0.03).

An analysis of tear film changes before and after treatment revealed significant alterations in tear functional parameters, reflecting improved tear film stability and secretory volume regulation, suggesting tear secretion normalization and functional equilibrium. Table 2 summarizes the results.

### 3.3. AC Patient Quality of Life Before and After BS Treatment

In terms of daily activities, the mean score was 8.3 times lower after treatment (*p* < 0.0001), with a minimum important difference of 2.2 points. Ocular symptoms (itching, tearing, and burning) decreased by 7.5-fold at the end of treatment (*p* = 0.004), with an MID of 1.4. Nasal symptoms (congestion, rhinorrhea, and sneezing) were reduced 5.5 times (*p* = 0.006) with an MID of 1.8 points. The emotional dimension (irritability, frustration, and annoyance) decreased 5.2-fold (*p* = 0.02), with a minimum important difference of 2.1. The sleep dimension diminished 2.8 times (*p* = 0.05), with a minimum important difference of 1.6. Other symptoms (fatigue, lack of concentration, or headache) decreased 3.8 times (*p* = 0.01). Similarly, practical problems (tissue use and eye rubbing) decreased 3.3 times, both with an MID of 1.4.

**Figure 2:**
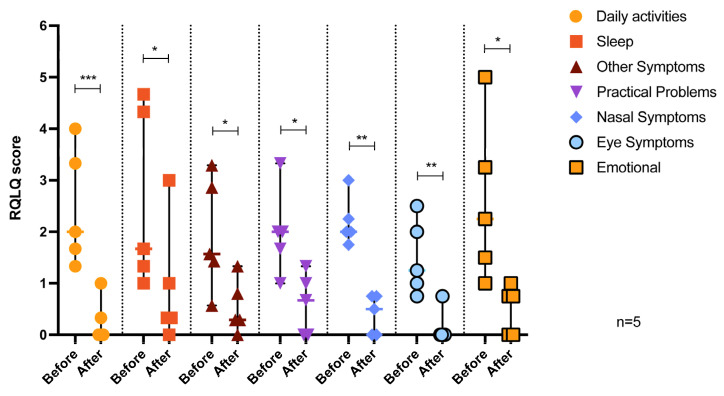
Scatter plots comparing Rhinoconjunctivitis Quality-of-Life Questionnaire (RQLQ) scores before and after BS treatment. Each point represents an individual patient’s measurement (n = 5) for the corresponding domain. Medians and 95% confidence intervals are also shown. Asterisks indicate statistically significant differences (* *p* < 0.05; ** *p* < 0.01; *** *p* < 0.001).

Finally, the total questionnaire score decreased 5.5 times (*p* = 0.001), with a minimum important difference of 1.7 points after 90 days of BS treatment (Figure 2).

### 3.4. Adverse Events Caused by BS

Adverse events were reported by patients and their parents (Table 3). All AEs were self-limited, and the vast majority resolved within 24 h of onset without any sequelae.

### 3.5. Changes in the Percentage of CD19^+^ IL-10^+^ Cells in Peripheral Blood

Peripheral blood samples were collected before and after BS treatment; they were analyzed by flow cytometry to determine the CD19^+^IL-10^+^ cell percentages and assess immunological changes. The proportion of CD19^+^IL-10^+^ cells increased 9.6-fold, from 3% at baseline (IQR, 2.5–3) to 29% (IQR, 22.5–32) at the end of treatment.

## 4. Discussion

In recent years, several research groups have explored the use of bacterial products as immunomodulatory therapies for treating allergic diseases. These formulations commonly include inactivated antigens from bacteria frequently associated with the upper respiratory tract and mucosal immune stimulation; their effects stem from their capacity to activate innate immunity and modulate downstream adaptive Th2 responses when administered sublingually [24]. In this work, commercially available bacterial components were selected based on the rationale that they exert immunomodulatory effects, particularly in respiratory allergic diseases, with the aim of offering a therapeutic alternative for patients with moderate-to-severe ocular allergy showing no identifiable triggering allergen by skin prick testing and who did not achieve clinical improvement despite standard ophthalmic treatment. It is important to note that the safety of bacterial product use has been well recognized in several studies examining pediatric populations with both infectious and allergic diseases [14,25,26].

In this exploratory study, we observed a significant reduction in ocular signs and symptoms after 3 months of treatment with a bacterial suspension, accompanied by improvements in ocular surface and tear film stability. From a pathophysiological perspective, controlling ocular surface inflammation is fundamental to restoring tear film homeostasis. Chronic conjunctival inflammation may compromise tear film stability, alter goblet cell function, and modify mucin production, thereby contributing to ocular surface deterioration. In this context, available evidence indicates that bacterial lysates increased the number of IL-10-producing T and B cells and inhibited IL-4 and IL-13 production [17,27,28]. IL-10 modulates the balance between Th1 and Th2 immune responses, promoting a more tolerogenic immune profile [29,30]. Limiting a Th2-inflammatory microenvironment could suggest better control of mucosal inflammation at the ocular surface, while bacterial products may be beneficial for controlling the inflammatory process. Consistent with these data, patients in our study showed an increase in CD19^+^IL-10^+^ cells after 3 months of BS treatment in the exploratory analysis performed (Supplementary Table S2). These findings support the potential benefit of bacterial products in modulating ocular inflammatory responses; however, further studies are needed to understand the mechanisms underlying these outcomes.

Regarding quality of life, we observed a significant improvement across all domains after BS treatment, particularly in daily activities, followed by eye symptoms. However, the minimum important difference was mainly seen in daily activities, followed by the emotional dimension. Similar to our study, a double-blind clinical trial in patients with allergic rhinitis treated with probiotics demonstrated significant reductions in ocular and nasal symptoms, along with improvements in quality-of-life scores [31], while the use of bacterial lysates improved clinical outcomes in a randomized controlled clinical trial in children with allergic rhinitis [32]. These findings support the concept that the immunomodulatory effects of bacterial-based interventions are mediated through immune crosstalk pathways rather than by the pathogenic nature of the bacterial components themselves or by the administration route. In biological systems, this is not an isolated effect. It is well established that allergic inflammatory conditions, such as asthma, may engage mechanisms similar to those involved in tumor cells, potentially promoting changes in organs distant from the primary site through alterations in the microenvironment [33,34]. A comprehensive evaluation of BS effects on the ocular surface will require the integration of complementary approaches, including the assessment of miRNAs, cytokine profiles, and immune signaling pathways, as well as their relationships with the ocular microbiome and genetic background. Such an integrative approach may help elucidate the mechanisms through which BS therapy and other immunomodulatory interventions could influence the ocular surface in allergic inflammation [35,36]. In this context, the bacterial suspension used in the present study should be interpreted as an inactivated antigenic stimulus capable of modulating mucosal and systemic immune responses, rather than as a pathogen-driven intervention.

The observed changes in TBUT and the Schirmer test may reflect an improvement in tear-film stability and ocular surface homeostasis. One possible explanation is that reduced ocular surface irritation and inflammation decreased reflex tearing, while improvements in tear-film composition enhanced its stability and reduced evaporation. Together, these effects may promote epithelial integrity and a more balanced ocular surface microenvironment [37]. However, because tear-film composition was not directly assessed, this mechanistic interpretation remains hypothetical. The improvements in ocular surface and tear film stability observed in the patients who received BS treatment may also explain the positive impact on quality of life via reduced ocular inflammation and eye symptomatology [38,39]. Future studies incorporating measurements of tear osmolarity, lipid-layer thickness and composition, mucin expression, and inflammatory mediators are needed to clarify the mechanisms underlying these clinical findings.

Bacterial-based treatments for several diseases have generally demonstrated favorable safety profiles in most clinical trials (controlled and non-randomized), with predominantly mild and transient adverse events, including gastrointestinal discomfort, abdominal distension, and headache [40,41]. Consistent with that evidence, in our study, the most frequent adverse events were also gastrointestinal; all patients described them as mild, tolerable, and self-limited, resolving on the same day of onset, which reinforces the adequate tolerability of this intervention.

Despite the favorable outcomes observed in our study, several limitations should be considered when interpreting these findings. First, the small sample size limits the statistical power and precision of the analyses, increases the risk of unstable estimates, and restricts the generalizability of the results to broader patient populations. In addition, the open-label, nonrandomized, uncontrolled pre–post design prevents the observed changes from being attributed exclusively to BS therapy. Without a control group, the potential contributions of observer bias, placebo effects, regression to the mean, natural fluctuations in disease activity, and the continued use of conventional ophthalmologic treatment cannot be excluded. Therefore, the statistically significant findings, including the exploratory correlations between immunological and clinical outcomes, should be interpreted cautiously and regarded as hypothesis-generating rather than confirmatory evidence. In addition, most patients included were male, which may have influenced the observed outcomes, as sex-related biological and immunological differences can affect both disease expression and treatment response. The predominance of boy participants should therefore be considered when interpreting the results and extrapolating them to more diverse populations. Long-term follow-up was challenging due to variability in patient availability for subsequent evaluations, which may restrict our ability to determine whether the observed clinical and immunological benefits are sustained over time. Nevertheless, the structured evaluation of clinical outcomes, together with the systematic assessment of immunological parameters, provides relevant preliminary evidence supporting the potential role of BS as a complementary therapeutic strategy in allergic conjunctivitis. Future studies with larger sample sizes, a control group, and longer follow-up periods will be necessary to confirm its clinical efficacy and to further elucidate the underlying immunomodulatory mechanisms, as well as the characteristics of the bacterial suspension used in this study, which contained mainly pathogenic bacteria.

Although local ocular immune mechanisms were not evaluated in this study, we cannot exclude the possibility that these patients represent a phenotype of local allergic conjunctivitis characterized by the absence of detectable systemic sensitization on skin testing [42]. In this subgroup, BS therapy may represent a potential therapeutic approach when no causative allergen can be identified. However, this hypothesis requires confirmation through local allergen-specific assessments and adequately designed controlled trials.

Overall, the preliminary evidence presented in this proof-of-concept study suggests that bacterial suspension treatment could be associated with significant improvements in clinical symptoms, reduced ocular surface inflammation, and improved quality of life, while maintaining a high safety profile. From an immunological perspective, the observed modulation could be mediated by CD19^+^ IL-10^+^ cells. These preliminary findings may also provide the basis for exploring BS as a novel complementary immunomodulatory strategy, particularly for patients who show an inadequate response to conventional ophthalmologic treatment.

## Figures and Tables

**Table 1:** Demographic characteristics.

Variable	Median (IQR)
Age (years)	8 (7–9)
Peripheral blood eosinophils (%)	2 (1–11.2)
Total serum IgE (IU/ mL)	73.2 (43.3–127.5)
Serial stool examination (3 samples)	Negative in all patients
Skin prick test (3 samples)	Negative in all patients

IQR: interquartile rank.

**Table 2:** Ophthalmological evaluation before and after BS treatment.

	Value Baseline Median (IQR)	Value After BS Treatment Median (IQR)	*p*-Value (Wilcoxon)
TBUT (sec)	5 (4.0–6.5)	9 (7–10)	0.002
Schirmer I (mm)	29 (17.5–35)	12 (11–14)	0.004

IQR, interquartile range; TBUT, Tear Break-Up Time.

**Table 3:** Adverse events *.

MedDRA Preferred Term (PT) ID PT	Causality	Severity	Day of Treatment Onset	Resolution
Diarrhea 10012727	Possible	Mild	3	Resolved without sequelae
Nausea 10028813	Possible	Mild	3	Resolved without sequelae
Abdominal pain 10000087	Possible	Mild	1	Resolved without sequelae
Vomit 10047700	Possible	Mild	30	Resolved without sequelae
Diarrhea 10012727	Possible	Mild	63	Resolved without sequelae
Nausea 10028813	Possible	Mild	1	Resolved without sequelae
Abdominal pain 10000087	Possible	Mild	1	Resolved without sequelae
Dysgeusia 10013911	Possible	Mild	3	Resolved without sequelae
Weight loss 10047900	Unlikely	Mild	30	Resolved without sequelae at day 40.
Abdominal pain 10000087	Possible	Mild	1	Resolved without sequelae

* More than one adverse event occurred in the same patient.

## Data Availability

All data related to this study are available in the supplementary tables.

## Supplementary Materials

The following supporting information can be downloaded at https://www.mdpi.com/article/10.3390/jcm15155962/s1, Table S1: Individual results before and after bacterial suspension treatment. Table S2: Bregs correlation.

## Abbreviations

The following abbreviations are used in this manuscript:

BS	Bacterial Suspension
AC	Allergic Conjunctivitis
IL	Interleukin
IQR	Interquartile Rank
RQLQ	Quality-of-Life Questionnaire on Rhinoconjunctivitis
CD	Cluster Differentiation
MID	Minimum Importance Difference
DECA	Consensus Document on Allergic Conjunctivitis
TBUT	Tear Break-Up Time
